# Besieging the Tumoral Sites: Could It Be an Alternative Therapeutic Strategy in Ductal Pancreatic Adenocarcinoma?

**DOI:** 10.7759/cureus.10909

**Published:** 2020-10-12

**Authors:** Mohamed Islam Delma

**Affiliations:** 1 Internal Medicine, Colonel Chaabani Hospital, El Menia, DZA

**Keywords:** desmoplasia, granuloma, macrophages, pancreatic ductal adenocarcinoma, immune checkpoint inhibitors

## Abstract

Pancreatic ductal adenocarcinoma is characterized by its high morbidity, and curative drugs are still lacking. In addition to immunotherapy, other molecular targeted therapeutics, such as stroma depleting agents, have been evaluated, given the abundant desmoplastic stroma that is considered a protective shield for tumor cells. However, the unexpected poor outcome has raised the debate on whether desmoplasia promotes or restrains tumor cell spread. After reviewing these key points in this paper, an approach taking advantage of desmoplasia and immune cells to besiege the tumoral sites will be proposed. Based on the available literature, the feasibility and potential limitations of this strategy will be discussed.

## Introduction and background

Pancreatic cancer is among the leading causes of cancer-related mortality. Pancreatic ductal adenocarcinoma (PDAC) and its variants are the most frequent type, representing 85% - 90% of all pancreatic neoplasms [1]. Surgery is the cornerstone of management for eligible patients. The recourse to adjuvant therapy, such as chemotherapy, is needed, given that the five-year survival after curative surgery alone is only about 8% - 15% [1]. Chemotherapy is also indicated for patients ineligible for curative surgery. Gemcitabine served as a reference, to which new regimens were compared. Fluorouracil, folinic acid, irinotecan, and oxaliplatin (mFOLFIRINOX) association, and gemcitabine plus nanoparticle albumin-bound paclitaxel (nab-paclitaxel), have become the preconised protocols in patients with favorable comorbidity profile [1-2]. 

Despite these combinations, the clinical response is still moderate, inciting for exploring new approaches, such as molecular-targeted therapy. This consists of blocking specific molecules and/or physiopathological mechanisms involved in PDAC development, such as the Ras signaling pathway, cancer stem cells, tumor immunomodulation, and desmoplastic stroma [2-3]. The two latter aspects will be reviewed in the next section. 

## Review

Immunotherapy 

Immunotherapy, such as immune checkpoint inhibitors (ICIs) and vaccines, aims to overcome tumor-induced immunosuppression and to boost the immune response. It has been applied to pancreatic cancer, given the importance of immunosuppression induced by tumor cells and its microenvironment [4]. Immune checkpoints are natural modulators of immune cells, but they are also expressed and exploited by some tumor cells. Immune checkpoint inhibitors targeting these molecules, notably cytotoxic T-lymphocyte-associated protein 4 (CTLA-4), programmed death-1 (PD-1), and its ligand programmed death-ligand 1 (PD-L1), have been developed to block the tumor-induced immunomodulation. However, the inefficacy of ICIs as monotherapy for PDAC has been attested to after a few clinical trials [5]. A possible reason could be the multitude of immune checkpoint molecules and pathways responsible for immunosuppression. This has led to a variety of combinations between different ICIs and with inhibitors of other immunomodulatory signals.

In a study published by the Canadian Cancer Clinical Trial Group, the addition of durvalumab (PD-L1 inhibitor) and tremelimumab (CTLA-4 inhibitor) to nab-paclitaxel or gemcitabine led to a 100% disease control rate, although the aimed median overall survival (med OS) had not been reached [6]. Nivolumab, an anti-PD-1, was associated with mogamulizumab, a CCR4 inhibitor that targets the regulatory lymphocytes (Treg), in a phase I clinical trial [7]. The combination showed an acceptable safety profile, and among 15 patients with PDAC, one had a partial response (PR), while five had disease stabilization. Durvalumab in association with oleclumab, a monoclonal antibody that binds to CD73 and inhibits the production of immunosuppressive adenosine, showed manageable side effects with partial response in two out of 20 patients [8]. A phase Ib/II randomized study (http://clinicaltrials.gov/ct2/show/NCT03611556) of more than 300 patients under different combinations between these two molecules and chemotherapy drugs is ongoing. ICIs have also been added to dendritic cell vaccines to enhance their efficacy, given the expression of PDL-1 on dendritic cells. There has been a stabilization of disease under this association in five of 10 patients that had previously failed the dendritic vaccine alone [9]. 

The reduced antigenicity of tumor pancreatic cells could also limit the outcome of immunotherapy. Therefore, chemotherapy and radiotherapy have been added, as the tumor lysis activity of these agents would cause a massive exposition of immune cells to tumor antigens. The association of gemcitabine, nab-paclitaxel, and pembrolizumab have achieved 100% of disease stabilisation, although the endpoint of > 15% complete response was not met. The median progression-free survival (PFS) and OS compare favorably with other regimens that were built on the basis of gemcitabine and nab-paclitaxel [10]. In the context of neoadjuvant therapy, the addition of pembrolizumab to chemoradiotherapy increased the percentage of patients who underwent surgery from 50% to 70% [11].

Targeting desmoplasia

Pancreatic cancer desmoplasia results from excessive production of the extracellular matrix (ECM), mainly collagens, by stromal cells such as cancer-associated fibroblast (CAF) and pancreatic stellate cells (PSC). Studies have suggested an underlying protective role of desmoplasia for tumor cells by preventing the infiltration of immune cells and the delivery of anticancer molecules [12]. In addition to some chemotherapy drugs, such as nab‐paclitaxel, depleting tumor stroma may also be achieved either by molecules inducing lysis of ECM, such as pegvorhyaluronidase alfa (PEGPH20) and CD40 agonists, or indirectly via drugs targeting CAFs, such as pirfenidone, pasireotide (SOM230), and Hedgehog (HH) pathway inhibitors [12]. An early phase II study showed a significant improvement in PFS with the addition of PEGPH20 to nab-paclitaxel/gemcitabine. This benefit was more marked in the subset of patients with hyaluronan-high tumor samples. However, the result was not replicated in a phase III trial, as the addition of PEGPH20 did not improve OS or PFS, even though it increased the objective response rate (ORR) [13]. As for CD40 agonists, which are activators of antigen-presenting cells (such as macrophages), a regional involution of the tumor stroma with a decrease in collagen I content after treatment with CD40 agonist was reported [14]. This effect was likely mediated by macrophages, given the absence of these modifications in mice depleted of systemic macrophages.

An example of drugs targeting CAF is the inhibitors of the HH pathway [15]. After encouraging experimental results and the absence of severe safety concerns, HH inhibitors have been tested in phase II clinical trials. Unfortunately, there was no improvement in PFS and OS. One clinical trial of saridegib, a HH pathway inhibitor, and gemcitabine even was discontinued, as the median survival was less than that of the historical one of gemcitabine alone [16]. One can argue that these inhibitors may have direct protumorigenic effects on tumor cells, which is less probable as HH signaling itself has many direct protumorigenic effects, such as the proliferation of cancer stem cells [17]. Even if we consider that autophagy induced by the blockade of HH has shown a protumorigenic effect in some tumors, this was not the case in pancreatic cancer [18]. This, therefore, leads back to the fundamental question of whether desmoplastic stroma restrains or promotes the tumoral process. Rhim and collaborators showed that in a mouse model of pancreatic cancer with deletion of sonic HH (a subtype of HH), there was a depletion of desmoplastic stroma as expected, but the tumors were more aggressive and exhibited undifferentiated histology, increased vascularity, and heightened proliferation [19].

The siege strategy

Based on these constatations, an alternative approach of besieging the tumoral lesions by reinforcement of the surrounding desmoplasia with immune cells, mainly macrophages, is proposed. The choice of macrophages is inspired by granulomatous reaction constated in some tumoral lesions. Some authors have found a better prognosis in some tumors [20-21], while others have not [22]. As we did not find a report concerning their presence in PDAC, some cases with favorable outcomes of granuloma associated with signet ring cell gastric carcinoma, which have also prominent desmoplasia, had been reported [23-24]. Macrophage attraction to tumoral sites is induced by the secretion of chemokines in the milieu, such as chemokine (C-C motif) ligand 5 (CCL5), which also has a role in granuloma formation [25]. It may be upregulated by some cytokines, such as interleukin 1 beta (IL-1β), interferon-gamma (IFNγ), tumor necrosis factor-alpha (TNFα), and interleukin 32γ (IL-32γ) [26-27]. IL-1β has been used for the treatment of solid tumors (given experimentally) reducing effects on tumor growth, and an increase of lymphocytes and inflammatory cells influx into the tumor [28]. Some phase I/II clinical trials have shown regression of tumors, although the response was not consistent. However, the use of IL-1β was limited due to high systemic toxicity, making this method unlikely to be applicable unless a method of local enhancement of IL-1β is implemented [28].

Another approach consists of amplifying the expression of CCL5 induced by IL-1β already present in the tumor microenvironment [29]. This could be realized through modulation of sphingosine-1-phosphate (S1P), which is an inhibitor of this induced expression via S1P receptor 2 (S1PR2) [30]. The existence of JTE-013, an experimental antagonist of S1PR2, would allow a preclinical verification of the concept. Currently, there is no S1PR2 antagonist included in a clinical trial phase. As for S1P antagonists, sonepcizumab had achieved a phase II clinical trial for unresectable renal carcinoma. Although the primary end-point concerning PFS was not met, the effect on OS is encouraging and may prompt further trials [31]. It would be interesting if patients with pancreatic cancer would be included eventually.

It is probable that macrophage infiltration would not be sufficient alone, and appropriate localization would also be needed [32]. In contrast to direct confrontation, the siege strategy would require that macrophages should be separated from tumor cells by some desmoplastic stroma to escape the immunomodulatory paracrine effects of these malignant cells. To achieve this, macrophages should be attracted or adhere to some constituents of the stroma, such as fibroblasts or ECM. The first option is challenged by studies reporting that interactions between CAF and macrophages also induce immunosuppression and tumor proliferation [33]. However, the study of Öhlund et al. showed that CAF has different roles according to the distance from neoplastic cells [34]. Therefore, it is interesting to evaluate the interaction between macrophages and fibroblasts at a localization relatively distant from tumor cells, notably if separated from them by some desmoplastic tissue. The chemoattraction of macrophages to fibroblasts could be induced by chemokines, such as chemokine (C-C motif) ligand 2 (CCL2), which is upregulated by cytokines, such as IL-1β, IFNγ, TNFα, and downregulated by others, such as transforming growth factor-beta 1 (TGFβ-1) [35-36]. The antitumoral effects of CCL2 on pancreatic cancer, in contrast to mammary tumors, and the encouraging results of TGFβ-1 inhibitors against PDAC in the phase II clinical trial facilitate the assessment of the effect of these inhibitors on the attraction of macrophages toward fibroblasts and the outcome of this colocalization [35, 37].

Concerning the interaction with ECM, macrophages are unlikely to adhere to major constituents of desmoplasia, such as type I collagen [38]. However, they adhere to collagen IV, a constituent of the basement membrane, raising the possibility of reinforcement of this membrane by macrophages. This interaction is mediated by the scavenger A receptor, which may be upregulated by the CD40 ligand [39-40]. However, the expected limitation would be the destruction of the basement membrane by macrophages themselves [41].

Apart from macrophage interactions with desmoplastic stroma, the immune siege could be realized through macrophage clustering. Its mechanism may be understood by studying the formation of granuloma and crown-like structures [42-45]. In these models, chemokines, such as IL-1β, CCL5, and osteopontin, have shown a role in the process. Osteopontin may be upregulated by estrogen receptor β (ERβ) modulation and vitamin D receptor (VDR) agonists [45-46]. Thus, evaluating the outcome of macrophage clustering in PDAC may be achieved through osteopontin upregulation via ERβ modulators or VDR agonists, a task advantaged by the fact that these drugs are already being studied for potential antitumoral effects against PDAC.

Targeting the tumoral vascularization to enhance the efficacity of the siege is rational, given its role in nutrients supply and metastatic dissemination, even in hypovascular pancreatic cancer [47]. After promising preclinical results, inhibitors of angiogenic factors, mainly, the vascular endothelial growth factor-A (VEGF-A), have been included in many phase II and III clinical trials in association with chemotherapy. However, most of these trials, notably, the phase III trials, failed to show an improvement in OS or PFS or differences in the ORR. Different explanations have been proposed, such as the limited efficacy of associated chemotherapy agents, poor drug diffusion, and the compensation between angiogenic factors. Concerning the latter one, the combination between inhibitors of two antiangiogenic elements, VEGFR-2 and EGFR, has indeed resulted in a significant amelioration of PFS [48].

In addition to the compensation between the angiogenic factors, the presence of de novo mechanism for tumor cell nutrition, consisting of basal microvilli in regions with less microvascular density (MVD), has been reported [49]. Still, the existence of experimental models where the antiangiogenic drugs have shown a reduction in angiogenesis may serve for the preclinical evaluation of these therapeutic contributions to the siege outcome [47]. Besides angiogenesis inhibition, it would be interesting to check if there were an attraction of lymphocytes to macrophage clusters and their subsequent role in barrier consolidation.

In summary, the outcomes of the two aspects of molecular targeted therapy (immunotherapy and desmoplasia depleting agents) have been reviewed. An additional approach of besieging PDAC by reinforcement of the desmoplasia barrier with immune cells (notably, macrophages) is proposed. Initially, potential methods to increase their number in the tumor microenvironment have been presented. After that, the necessity of appropriate localization and/or clustering was emphasized. Finally, possible potentialization of the siege by angiogenesis inhibitors and lymphocytes has been advocated. The feasibility and limitations of each step have been discussed, based on available literature. Eventually, reported cases of granulomatous reaction in PDAC would give hints about the outcome of some aspects of this approach, although experimental validation would be ultimately needed.

## Conclusions

In the absence of curative drugs for PDC, different approaches are being proposed. Some have been already tested, while others are still hypothetical. Depleting the stroma or taking advantage of it to besiege the tumoral sites are two opposite approaches, based on how desmoplasia is regarded - a protective shield or an enclosure for tumor cells. Experimental comparative data would have a response concerning the best strategy between them.
